# The Diagnostic Value of Clinical Sacroiliac Joint Pain Provocation Tests for Detection of Sacroiliitis Identified by MRI in Sampled Iraqi Patients with Inflammatory Back Pain

**DOI:** 10.31138/mjr.33.1.48

**Published:** 2022-03-31

**Authors:** Sami Salman, Farah Jaafar

**Affiliations:** 1 College of Medicine, Baghdad University, Baghdad, Iraq,; 2 College of Medicine, Al-Mustansiriya University, Baghdad, Iraq

## Abstract

**Background:**

Sacroiliitis has been considered as the keystone in the diagnosis of axial spondyloarthritis (SPA). The diagnosis can be challenging in early stages of sacroiliitis, as conventional radiographs may be normal. Pain provocative tests can be very helpful to give a clue whether sacroiliitis is present in suspected patient by reproducing the patient’s pain.

**Objective:**

To assess the validity of clinical sacroiliac joint pain provocation tests for sacroiliitis in comparison with magnetic resonance imaging (MRI).

**Patients and methods:**

A total of 65 patients were selected from cohort of patients with persistent inflammatory low back pain during their attendance to the Rheumatology Consultant Clinic at Baghdad Teaching Hospital. Data were collected using questionnaire and interview. Socio-demographic characteristics, disease duration and clinical data were recorded for all patients. Data from four different clinical examination tests (Flexion Abduction and External Rotation [FABER], direct compression, distraction, and lateral compression) with subsequent MRI findings of sacroiliac joint for each patient were analysed. Positive MRI findings were further subdivided into active and chronic lesions according to the new ASAS (association of spondyloarthritis international society) criteria of positive imaging in spondyloarthropathy.

**Results:**

A total of 65 patients were included in this study, age range 18–39 years, 69.2% were men. The prevalence of sacroiliitis in the study was 67.7% (43.1% bone marrow oedema, 18.5% erosions, 15.4% sclerosis, 6.2 % for each of effusion; ankyloses and capsulitis). In the whole study group, the highest association with sacroiliitis was for FABER test with sensitivity of 75% while the most specific one was the lateral compression test (71.4%). In men, sacroiliitis was associated with sacroiliac joint (SIJ) clinical tests assessed in multi-test regimens; significant association was found by combining direct compression and FABER test with sensitivity of 51% and specificity of 28.5%.

**Conclusion:**

This study identified the reliability of a number of clinical examination tests of SIJ, which increase as they are used in multi-test regimen rather than each one alone, this had a significant association for men only.

## INTRODUCTION

The sacroiliac joint (SIJ) is a diarthroidal joint with both hyaline and fibrocartilage. The function of the SIJ is to transmit the axial forces from the lower limbs to the spine.^1^ It is usually overlooked as a source of buttock and/or low back pain (LBP). It can present in variable ways; typically, it presents as gluteal pain.^2^ Sacroiliitis refers to the inflammation of the SIJ. Diagnosis is made with difficulty due to its anatomical position and the absence of optimal diagnostic method.^3^

Axial spondyloarthropathies (AxSpA) comprise a group of disorders that affect the axial skeleton. A characteristic feature of AxSpA is unilateral or bilateral sacroiliitis.^4,5^ Structural damage contributes to the morbidity of AxSpA, so it is mandatory to recognise and treat sacroiliitis early.^6^ Many patients with AxSpA, especially at early stages of the disease, can present atypically as acute, unilateral pain with radiation to the lower limbs, which may be mis-diagnosed as a mechanical pain.^7,8^ The diagnosis can be challenging in early stages of sacroiliitis, as conventional radiographs may be normal. Magnetic resonance imaging is an established tool to enable early recognition of sacroiliitis.^9,10^ It is superior to computed tomography (CT), scintigraphy and conventional radiography for early diagnosis of sacroiliitis.^11^ The study of Maksymowych et al. clarified the new definitions of positive MRI according to Assessment of SpondyloArthritis International Society (ASAS), in which it was divided into findings that indicate activity (BME, capsulitis, joint space enhancement, inflammation at site of erosion, and enthesitis) and those that indicates structural changes(erosion, fat lesion, fat metaplasia, sclerosis, ankyloses, and bone bud).^12^

## AIM OF THE STUDY

To assess the validity of clinical sacroiliac joint pain provocation tests for sacroiliitis in comparison with magnetic resonance imaging (MRI).

## MATERIALS AND METHODS

This descriptive study was conducted at the Rheumatology Unit of the Baghdad Teaching Hospital, in collaboration with the Radiology Department in the same hospital from December 2019 until September 2020. The study protocol was approved after review and official permission was obtained from the Ethics Committee in the Medical Department, College of Medicine, University of Baghdad, numbered 100 date 2-1-2020. Informed consent was taken from each participant. All the identifying information’s were concealed during statistical analysis. A total of 65 participants aged 16–40 years old were selected from a cohort of patients with persistent inflammatory low back pain during their attendance to the Rheumatology Consultant Clinic. They were evaluated and enrolled after fulfilling the inclusion and exclusion criteria.

### Inclusion criteria

Patients were included in the study if they have persistent inflammatory LBP for more than 3 months fulfilling ASAS 2010 criteria for diagnosis of inflammatory back pain which include any 4 of the following:

1. Age 16–40 years at the onset of pain.
2. Insidious onset.
3. Relieved by exercise.
4. No improvement with rest.
5. Night pain (second half of night).

### Exclusion criteria

1. Mechanical low back pain.
2. Nerve root compression signs.
3. Contraindication to MRI.
4. Pregnancy.
5. Refusal to participate in the study.

**Figure 1.**
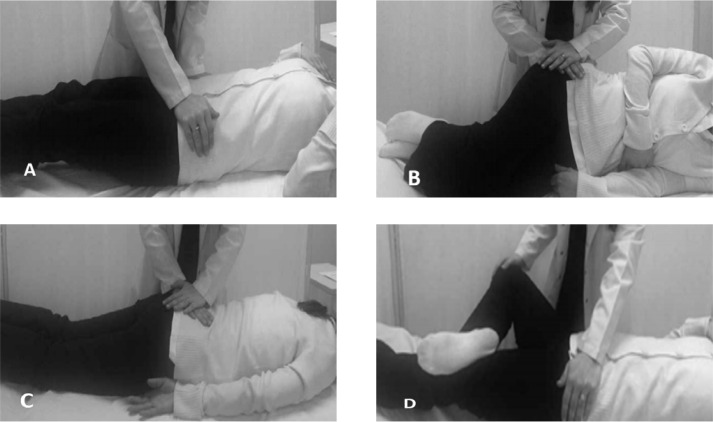
Manoeuvres of sacroiliac joint pain provocative tests^3^: (**A**) distraction test, (**B**) lateral compression test, (**C**) sacroiliac thrust test, (**D**) FABER test.

Each patient was examined for the following tests as shown in **Figure 1.**

#### Distraction test

The patient lies in supine position, slow and steady pressure is applied on each side of the pelvis.

#### Lateral compression test

Patient lying with the painful side facing up, knees are bent, and a pressure applied to the pelvis.

#### Sacroiliac thrust test

Direct SIJ compression in prone position.

#### Flexion Abduction External Rotation (FABER) test

Flexion Abduction External Rotation of the painful side, then the clinician places his or her hand on the pelvis of the opposite side and applies pressure to the flexed knee to exaggerate the motion further and this might elicit pain if the joint is inflamed.

## MRI PROTOCOL AND LESION DEFINITIONS

Each patient included in this study, regardless of the results of the clinical tests mentioned above, was sent for subsequent MRI of the SIJ. All MRI examinations were performed on a 1.5-T MR scanner (Philips Achieva/2011) using a four-channel phased-array body matrix coil. MRI scans included coronal T1-weighted turbo spin echo and short tau inversion recovery (STIR) sequences in coronal oblique and axial planes, angled parallel to the sacroiliac joint. The scan parameters for all sequences were as follows: 15–19 slices, 3mm slice thickness, 0.4mm inter-slice gap, and field of view 280–300mm. These were read by the same expert radiologist who had 12 years of experience in radiology and was blinded to all clinical information except the patients’ age and gender, as the request form was only to exclude sacroiliitis in each patient. The MRI was considered positive if meets ASAS criteria for active sacroiliitis which includes at least two hyper-intense lesions in the same slice or single hyper-intense lesion in at least two sequential slices and classified according to recently published EULAR update for defining a positive MRI.

## RESULTS

The study comprised 65 patients. All of them fulfilled the ASAS criteria for classification of inflammatory back pain (**Appendix 2**). The sociodemographic characteristics of patients are shown in **Table 3**. Of those 65 patients, 69.2% were men. Of those 65 patients, 67.7% of patients included in the study had sacroiliitis. The most prevalent MRI finding was bone morrow oedema (43.1%), while the least was capsulitis, enthesitis and ankyloses (6.2% for each).

**Table 3.** Diagnostic value of clinical sacroiliac joint pain provocative tests for sacroiliitis.

	**Total Study Sample**		**Men**		**Women**	
**Clinical test**	**Sensitivity**	**Specificity**	**Sensitivity**	**Specificity**	**Sensitivity**	**Specificity**
Distraction test	54.5	33.3	51.8	33.3	58.8	33.3
Direct compression	70.5	19.0	63	11.1	82.4	66.7
Lateral test	31.8	71.4	22.2	66.7	47.1	100
FABER test	75.0	19.0	70.3	16.7	82.4	33.3
Only One test	90.9	9.5	25.9	16.7	17.6	-
Only Two tests	68.2	23.8	18.5	11.1	17.6	33.3
Only three tests	50.0	38.1	33.3	33.3	17.6	33.3
All four tests	22.7	71.4	11.1	33.3	41.2	-
FABER+DCT	51	28.5	44.4	22.2	70.6	66.7

FABER: Flexion Abduction External Rotation.

In whole study group, the most prevalent clinical test was FABER test (76.9%) with the lateral compression test was the least prevalent. The most sensitive test was FABER test (75%) and direct compression test (70.5). Most specific test was lateral compression test with specificity of 71.4%, while the least was for FABER and DCT (19% for each).

By using multi-test approach, only 24.6% had all 4 clinical examination tests positive with the greatest sensitivity was for the combination of two tests (68.2%), while the most specific one was combination of four tests (71.4%). Notably, using only one test was the most sensitive but the least specific.

The clinical tests were evaluated on gender bases as well. In men group, 61.4% had sacroiliitis with significant P value of 0.047. For whom the highest association of sacroiliitis was with FABER test 70.4%. Combination of direct compression test and FABER tests had significant association with sacroiliitis with P value of 0.027 this was found in men group only.

In the group with women, the most sensitive tests were the DCT and FABER tests (82.4 % for each). Lateral compression test was the most specific one (100%).

## DISCUSSION

To the best of our knowledge, this is the first study that assesses the association between clinical sacroiliac joint pain provocation tests and sacroiliitis detected on MRI according to the new ASAS definition of positive MRI in AxSpA. Sacroiliitis is a core manifestation of axial spondyloarthritis (AxSpA). In western countries, the prevalence of AxSpA is about 1%.^13^ In Iraq, the estimated prevalence of ankylosing spondylitis (AS) is 0.13%.^14^ Still, the estimated diagnostic delay for axSpA is 8–14 years.^15,16^

This descriptive study revealed gender-based difference in the findings. Clinical tests of SIJ have been assessed in this study, significant association with sacroiliitis was found in men with p value of 0.047. Same was found in Fernandez-Sueiro et al. study.^13^

**Table 1.** Socio-demographic characteristics of the study population.

			**No**	**%**	
Age (years)		18–29	26	40.0	
30–39		39	60.0		
Mean±SD (Range)		30.4±6.2 (18–39)			
Gender		Male	45	69.2	
Female		20	30.8		
BMI (Kg/m^2^)		Normal (18.5–24.9)	27	41.5	
Overweight (25–29.9)		19	29.2		
Obese (=>30)		19	29.2		
		Mean±SD (Range)	27.3±4.5	(21.2–35.4)	
Marital status		Single	17	26.2	
Married		48	73.8		
Smoking		Smoker	28	43.1	
Passive smoking		9	13.8		
Non-smoker		28	43.1		
Level of education		Illiterate	2	3.1	
Primary		17	26.2		
Intermediate & Secondary		20	30.8		
College & higher		26	40.0		
Occupation and spinal load		Heavy	13	20.0	
Light		31	47.7		
Not		21	32.3		
Disease duration (months)	<12 months	13	20.0		
	12–22	16	24.6		
	23–35	10	15.4		
	36–47	5	7.7		
	48–59	5	7.7		
	=>60 months	16	24.6		
	Mean±SD (Range)	30.4±24.1 (3–72)			
Chronic diseases		Yes	18	27.7	
No		47	72.3		

N: number; SD: standard deviation; BMI: body mass index; Kg/m^2^: kilogram per square meter.

In the male group, clinical examination tests had a significant association with sacroiliitis, especially when combining direct compression and FABER tests with P value of 0.027. This gender-based variation in findings is probably due to higher prevalence of AxSpA in men, as it is known to be three times more common in men than women.^17^ Same findings were reported by the Arnbak et al. study,^11^ which was conducted on 454 patients. The prevalence of sacroiliitis in the Arnbak study was 5%, while it was 67.7% in the current study; this can be explained by the difference in the inclusion and exclusion criteria used in both studies.

In this study, the most sensitive and accurate clinical test was the FABER test, while the most specific one was the lateral compression test.

Only 16 patients (24.6%) had all four clinical tests positive. The accuracy was only 38.5%, this might be explained by number of factors that might affect a patient report and reaction to the clinical tests, as patient expectations, fear, approval seeking, psychological distress, and narrative styles as Carragee et al.^18^ study concluded.

**Table 2.** Prevalence of positive MRI findings in the study population.

MRI findings		No	%
sacroiliitis		44	67.7
Normal MRI		21	32.3
Bone marrow oedema		28	43.1
Capsulitis		4	6.2
Enthesitis		10	15.4
Erosion		12	18.5
Sclerosis		10	15.4
Effusion		4	6.2
Ankyloses		4	6.2
Number of positive MRI findings	None	21	32.3
	One	22	33.8
	Two	16	24.6
	Three	6	9.2
	Four	-	-

MRI: magnetic resonance imaging

In the current study, most SI joint clinical tests have limited reliability and validity on their own. While a multi-test regimen is a reliable method, Karolina et al. systematic review^19^ stated that the use of combined tests is of good diagnostic validity. Also, the Telli et al. study concluded the same, and that these tests can be used instead of unnecessary invasive diagnostic SI joint procedures.

In this study, these clinical tests are more sensitive than specific which is comparable to McGrath et al.^20^ study which stated that these tests are non-specific, as it causes pain provocation for variety of structures near the SIJ, such as the posterior sacro-coccygeal neurovascular plexus, ligaments, and muscles.

Regarding type of the active inflammatory lesion, this study revealed that, the most prevalent active lesion was bone marrow oedema (BME) of 43.1% and of the chronic lesions were erosions (18.5%). These findings are comparable to Maksymowych et al. study which was conducted on 278 MRI scans from the ASAS classification cohort by global assessment (lesion present/absent), and detailed scoring (inflammation and structural).

On the other hand, inflammation may fluctuate over time in SpA patients and 10% of patients with confirmed SpA on clinical ground may still have a normal MRI. Chronic changes on MRI, such as fatty degeneration, were documented but not considered in the analyses, because their value has not yet been defined precisely.^21^

There are some limitations in this study: this descriptive study in a single-centre, small sample size with time limit, and the financial barrier to perform MRI on every patient with inflammatory LBP. Many patients who were residents of cities that are distant from the study centre were not involved, as they stated the difficulties of coming back for the follow up for imaging; all these factors affected our sample size. Spinal MRI not included in the study as a further step to assess those with highly suspicion of SPA with negative SIJ MRI.

The main obstacle in this study was the COVID-19 pandemic which interfered with the collection of a larger number of patients to evaluate due to quarantine which began after a short period of starting data collection. However, this is the first study in Iraq that evaluated the diagnostic value of SIJ pain provocation tests as a potential tool to assess for the presence of sacroiliitis according to the new ASAS definition. They are simple, rapid, inexpensive, and non-invasive, and can be applied in everyday clinical practice.

## CONCLUSION

In conclusion**,** the reliability of clinical SIJ pain provocation tests increase as they are used in combination rather than either alone. The most sensitive of all four clinical tests was the FABER test, while the most specific was the lateral compression test. Significant association was found in men by combining direct compression and FABER tests, this had highlighted that the use of SIJ tests for prediction of sacroiliitis may be optimized by gender-separate analyses.

## Appendix 1.

ASAS criteria (2010) for classification of inflammatory back pain:

1. improvement with exercise
2. pain at night (at second half of night).
3. insidious onset
4. age at onset <45 years
5. no improvement with rest.

At least four out of these five parameters are needed to be fulfilled for classification of inflammatory back pain. Criteria had a sensitivity of 77.0% and specificity of 91.7%.^7^

Reference: Sieper J, van der Heijde D, Landewé R, et al. New criteria for inflammatory back pain in patients with chronic back pain: a real patient exercise by experts from the Assessment of SpondyloArthritis international Society (ASAS). Ann Rheum Dis 2009;68(6):784–788. doi:10.1136/ard.2008.101501

## Appendix 2.

The diagnostic value of clinical sacroiliac joint pain provocation tests for detection of sacroiliitis identified by MRI in sampled patients with inflammatory back pain.

Date
Name, Phone.
Consent:
Occupation:
Age:
Sex:
Marital status:
BMI (Wt/Ht):
Smoking status: Current, ex-smoker, never, passive
Alcohol:
Education level: Illiterate, primary, secondary
College (postgraduate):
Site of pain:
Duration of symptoms:
Aggravating factors/relieving factors:
Presence of comorbidities: non, 1, >1
Examination:
Distraction test
  SIJ thrust (direct compression)
  Lateral compression test
  FABER test
  MRI finding
